# Adsorption of Malachite Green with Sodium Dodecylbenzene Sulfonate Modified Sepiolite: Characterization, Adsorption Performance and Regeneration

**DOI:** 10.3390/ijerph16183297

**Published:** 2019-09-07

**Authors:** Jian Yu, Lirong Zhang, Bin Liu

**Affiliations:** 1 Department of Water Engineering and Science, College of Civil Engineering, Hunan University, Changsha 410082, China; 2 Department of Chemical Engineering, Process Engineering for Sustainable Systems (ProcESS), KU Leuven, Celestijnenlaan 200F, B-3001 Leuven, Belgium

**Keywords:** malachite green, sodium dodecylbenzene sulfonate, sepiolite, organically modification, adsorption

## Abstract

The adsorption of malachite green (MG) onto sodium dodecylbenzene sulfonate (SDBS)-modified sepiolite was investigated with respect to pH, oscillation rate, MG dosage and adsorbent dosage. The modification condition and modified sepiolite characterization were examined. The conditions of 100% cation exchange capacity (CEC), pH value of 9, contact time of 60 min and 25 °C were deemed as the optimal conditions. The interlayer spacing of sepiolite was expanded and the surface hydrophobicity improved due to the entering of SDBS into the interlayer structure of the sepiolite ore. This is probably the reason for its adsorption enhancement. The adsorption of malachite green by organic sepiolite is in line with the quasi-secondary kinetic model. The results from the regeneration procedure suggest that a superior regeneration property obtained with 0.2 mol/L HCl concentration.

## 1. Introduction

As mixtures of wastewater, printing and dyeing wastewater contain dyes, slurries, acids, bases, inorganic salts, oil agents. fiber impurities, etc. [1,2]. With the properties of a large water content, high organic matter content, high alkalinity, complex water quality, and biological refractory, dye treatment technology has been considered by researchers and become a nodus in the field of industrial wastewater treatment [3]. The chroma, which contains amine compounds, nitro and copper, zinc, chromium, arsenic and other heavy metal ions, has a high toxicity to organisms (it is carcinogenic, teratogenic, and mutagenic) [4].

Adsorption is widely applied in wastewater pretreatment and advanced treatment, and it has merits of simple operation conditions and low operating cost [5]. This method uses a powdery or granular porous material mixed with wastewater to remove harmful substances by the adsorbent surface of the porous medium. The adsorbent can be classified into the inorganic adsorbent (including carbon material, oxide, industrial waste, and clay mineral), the biomass adsorbent (including agricultural waste and the organic synthetic biosorbent), and the mixed adsorbent (nanocomposite pectin thorium(IV) tungstomolybdate (Pc/TWM), and starch/poly(alginic acid-cl-acrylamide) nanohydrogel) [6]. The common features of various adsorbents are: A large specific surface area, a certain surface structure, a suitable pore structure, a strong selective adsorption capacity for adsorbate, no chemical reactivity with the medium, a convenient preparation process, easy regeneration, good mechanics strength [7]. A novel adsorbent with a low price and a high regeneration rate has engineering application value.

Malachite green (MG) is a kind of emerald crystal with a metallic luster that is widely employed in ceramics, textiles, leather, food dyeing and cytochemical dyeing areas [8,9]. Due to the presence of a double bond on the carbon which connects three phenyl groups, it is easily metabolized in aquatic organisms to form a colorless leuco malachite green. Since the electric potential of MG is close to certain enzymes constituting cells, MG may compete with the enzyme site during cell division and thus hinder the formation of normal protein peptides, thereby exerting certain antibacterial and anti-virus effects. The functional group of malachite green is triphenylmethane, which contains a methylene and methine group bonded to the phenyl group and that has high reactivity potential with the benzene rin and can form a trityl radical. Furthermore, malachite green can attenuate the activity of glutathione-S-transferase, which can alter the oxygen pressure in organ tissues, resulting in abnormal apoptosis in cells.

The standard chemical formula of sepiolite crystals is Mg_8_Si_12_O_30_(OH)_4_(OH_2_)_4_·8H_2_O, and belongs to the S monoclinic or orthorhombic system, which is a layer and chain transition structure [10]. A layer of the octahedron is sandwiched between the two layers of siloxane tetrahedron. Since the tetrahedron is continuous, the oxygen at the apex angle of each siloxane intersects and is arranged in a 2:1 chain. The three-dimensional structure and the silicon–oxygen bond pull the thin chain together so that sepiolite has a prolonged special crystal form and the particles are rod-shaped. The surface of sepiolite is rich in a large number of acid–base centers, exhibits a strong polarity, and a exhibits strong adsorption capacity for polar substances [10]. A coupling agent or a surface treatment agent can improve the dispersibility and cohesiveness of sepiolite. The organic modifier could esterify, condense, or etherify with the Si–OH group of sepiolite, thereby introducing carbon chains of the crystal structure. The organic modification of sepiolite could improve its surface hydrophobicity, reduce its surface energy, and enlarge the cross-sectional area of its pores [11]. Hunan Province, China, has over 20% of the world’s sepiolite reserves, and the price of sepiolite ore is much lower than other common adsorbents such as activated carbon [11]. Hence, developing a high efficiency sepiolite-based adsorbent is of significant value.

In this work, sodium dodecylbenzene sulfonate was utilized for the organic modification of sepiolite. Firstly, the optimization of the modification process was investigated, and the optimized sepiolite was characterized with the Brunner–Emmet–Teller (BET) measurement, X-ray diffraction spectroscopy (XRD, Fourier transform infrared spectroscopy (FT-IR) and scanning electron microscopy (SEM). Moreover, the impact of pH, oscillation rate, MG dosage and adsorbent dosage on adsorption performance was comprehensively discussed. Additionally, the adsorption thermodynamics and kinetics analyses, as well as a regeneration test, were employed.

## 2. Materials and Methods

### 2.1. Materials

The target substance, malachite green (MG), was bought from Kermel Company, Tianjin, China. MG was centrifuged at the speed of 10,000 r/min for 10 min, and the supernatant was employed for the adsorption test. The sepiolite was supplied by Guangda Sepiolite Company, Changsha, China. Detail information of MG and sepiolite can be found in the support information. Other chemical agents, including sodium dodecylbenzene sulfonate (SDBS), NaOH, H_2_SO_4_ and BaCl_2_, were at least of analytical grade unless otherwise stated.

### 2.2. Preparation of Organically Modified Sepiolite

To prepare the organically modified sepiolite, the ore was firstly purified by settlement and then sieved with 200 mesh before the modification process. Various dosages of anionic surfactant and 10 wt% sepiolite were utilized for the wet modification method. After a complete mixing, the modified sepiolite was drying at 75 °C and then milled through a 200 mesh screen. Based on the cation exchange capacity (CEC) of original sepiolite (the CEC of original sepiolite was 101.94 mmol/100 g), the dosage of SDBS equivalents to 20%, 40%, 60%, 80%, 100%, 150%, 200% CEC was selected for the modification. The effects of pH, mixing duration and mixing temperature on the property of modified sepiolite were also investigated.

### 2.3. Characterization

The specific surface area of the adsorbent was measured by a Brunner–Emmet–Teller (BET) measurement (QuadraSorb SI, Quantachrome, Boynton Beach, FL, U.S.) [12]. X-ray diffraction spectroscopy (XRD) was used with an X-ray diffraction spectrometer (D8-Advance, Bruker, Karlsruhe, Germany) [13]. The tube pressure was 20 KV, the scanning range was 5–60°, and the scanning speed was 0.02°/0.2 s. The substance contained therein was determined based on the diffraction database. Fourier transform infrared spectroscopy (FT-IR) was detected with a Fourier transform infrared spectrometer (Spectrum One B, Perkin Elmer, Waltham, MA, USA), and the scanning range was 4000–500 cm^−1^ [13]. To observe the appearance features, a scanning electron microscope (Quanta 200, FEI, Hillsboro, OR, USA) was employed to observe the features of modified sepiolite [14].

### 2.4. Adsorption Test

To determine the quality of the adsorption procedure, the decolorization ratio and adsorption amount according to the Lambert–Beer law were employed, and the calculation method is shown in Equations (1) and (2) [15].

(1) $$\eta \%=\frac{C_{0}-C}{C_{0}}\times 100\%$$

(2) $$q=\frac{\left(C_{0}-C\right)V}{1000m}$$

where *ƞ*% is the decolorization ratio of MG, *C*_0_ and *C_e_* are the initial and final concentrations of MG, *q* is the adsorption amount, *V* is the solution volume, and *m* is the weight of the adsorbent.

In this study, the impact of pH, oscillation rate, adsorbent dosage and MG dosage on the adsorption performance was comprehensively investigated. The kinetic experiments were carried out at pH ranging from 1.5 to 8.5, an oscillation rate ranging from 80 to 200 r/min, an adsorbent dosage ranging from 1 to 5 g/L, and an MG dosage ranging from 100 to 500 mg/L.

Many simulation methods were employed to investigate the adsorption mechanism, including isotherm, kinetics and some combined approaches [16,17]. To further understand the adsorption law, the adsorption isotherm and adsorption kinetics were discussed in detail. Two classical adsorption equations, the Langmuir equation and the Freundlich equation, were used for the data fitting. In addition, the pseudo-first-order adsorption and pseudo-second-order adsorption equations were employed as fitting models to determine the kinetics law [18]. The intraparticle diffusion kinetics equation and the apparent adsorption activation energy equation were used to predict adsorption kinetics and to evaluate the minimum activation energy, respectively. All the equation fittings were calculated using MATLAB software (MathWorks, Natick, MA, USA).

### 2.5. Acid Regeneration Test

During the regeneration test, the adsorbent, which had reached the adsorption equilibrium, was immersed into 30 mL of an HCl solution. The mixture was shaken at a normal temperature for a certain period of time, and then the adsorbent was filtered out and dried at 45 °C to obtain a regenerated organic adsorbent. 1 g/L of the regenerated adsorbent was dosed into 100 mg/L of the MG solution (50 mL) for 2 h at the conditions of a 170 r/min oscillation rate and a pH of 3.5. The effects of different hydrochloric acid concentrations, reaction durations and reproduction times on regeneration performance were investigated.

## 3. Results and Discussion

### 3.1. Optimization of the Modification Process

**Figure 1:**
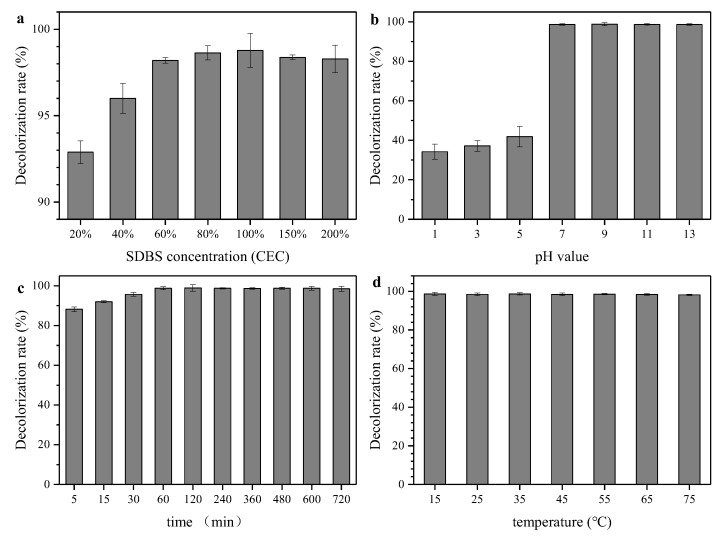
Effect of sodium dodecylbenzene sulfonate (SDBS) concentration, pH, contact duration and temperature on the sepiolite modification performance. To evaluate the decolorization rate and adsorption amount, 1 g/L of modified sepiolite under different modification processes was dosed to a 50 mL malachite green (MG) solution (100 mg/L) with a pH value of 3.5. Each test was sampled for the decolorization rate and the adsorption amount determination after being oscillated in a 170 r/min oscillation box for 2 h under a constant temperature (25 °C). In (**a**), a wet modification process was conducted for 60 min under an initial pH value of 9 and a temperature of 25 °C; in (**b**), a wet modification process was conducted for 60 min under 100% cation exchange capacity (CEC) and 25 °C; in (**c**), a wet modification process was conducted under 100% CEC, an initial pH value of 9 and a temperature of 25 °C; in (**d**), a wet modification process was conducted for 60 min under an initial pH value of 9 and 100% CEC.

As shown in Figure 1, the decolorization and adsorption amount of the modified sepiolite with various CEC ratios, pH, contact durations and temperatures were investigated. It can be observed from the figure that the decolorization rate and adsorption amount of organic sepiolite to MG improved with the increase of the CEC ratio from 20% to 100%. When the SDBS concentration was equivalent to 100% CEC, the modified sepiolite ore performed with optimal decolorization and adsorption effects, and the continued increase of the SDBS ratio slightly decreased its effect on adsorption. This probably because of the “dual layer” formed by excessive SDBS on the surface of the sepiolite with van der Waals force. Additionally, the hydrophilic group of the outer layer faces outward, which in turn leads to an enhanced hydrophilicity and weakened lipophilicity, and it results in the reduction of MG adsorption. Figure 1c,d presents the decolorization and adsorption amount with different pH values. The adsorption amount was lower than 42 mg/g under the modification process with acid conditions. This is probably due to the abundant H^+^ adsorbed by the sepiolite surface under acidic conditions, and H^+^ could exchange Mg^2+^ and Si^4+^ from sepiolite. Hence, the electrical repelling between the colored cations and sepiolite surface could hinder the dye adsorption. On the other hand, H^+^ was also ionized with the anionic surfactant, and the anion binding weakened the effect of sepiolite modification. The adsorption performance was significantly promoted under neutral and alkaline conditions [19]. The adequate contact of SDBS and sepiolite was beneficial to the adsorption performance. However, the enhancement of adsorption with contact time was feeble when the duration was over 60 min. Figure 5d shows that the factor of temperature had little effect on the SDBS modification process. When the temperature increased from 15 to 75 °C, the decolorization rate was maintained at around 98% and the adsorption amount was up to 97.8 mg/g. This may due to the porous property of sepiolite, which similar to the zeolite molecular sieve [11]. It also has abundant basic groups and acidic centers on its surface. The strong polarity of sepiolite was conducive to polar substances adsorption such as SDBS. On the other hand, the Si–OH group on the surface of sepiolite showed good affinity to the surfactant. After a comprehensive investigation of modification conditions, the 100% CEC, pH value of 9 (the pH value of the initial SDBS solution and sepiolite was near 9), contact time of 60 min and temperature of 25 °C were deemed as the optimal conditions, and the modified sepiolite under these condition was utilized for the characterization and further adsorption tests.

### 3.2. Characterization of Modified Sepiolite

**Figure 2:**
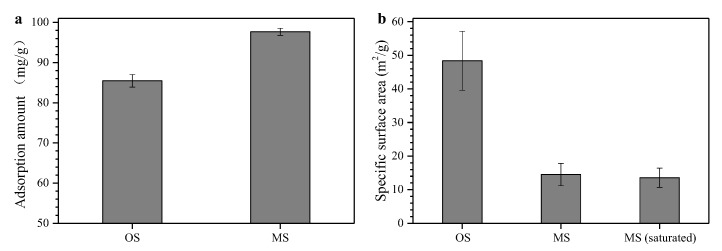
Comparison of MG adsorption amount (**a**) and specific surface area (**b**) of different adsorbents. In the abscissa label, PAC represents powder activated carbon, OS represents original sepiolite, MS represents modified sepiolite, and MS (saturated) represents the modified sepiolite after adsorption saturation.

It can be seen from Figure 2a that the adsorption capacity of modified sepiolite for MG was much higher than that of sepiolite ore. The adsorption capacity obviously improved from 85.46 to 97.67 mg/g after SDBS modification. This may be due to the adsorption of the macromolecular organic groups of SDBS on the surface of sepiolite to form a sepiolite-organic surfactant complex, which improved the hydrophobicity of sepiolite and the sepiolite modified to a hydrophobic organic sepiolite [20]. When the organic dye was adsorbed, the surface energy was greatly reduced, and the ability to remove organic dyes was enhanced according to the principle of “similar compatibility.”

Figure 2b shows that the modified sepiolite had a lower specific surface area than the sepiolite ore. This probably because the surfactant entered or adhered to the surface of the sepiolite after modification. Some adsorption sites on the surface of the sepiolite may have been cleaned, and the specific surface area was reduced. On the other hand, less than 1 m^2^/g specific surface area decreased after the saturated adsorption test, which suggests that the total specific surface area was not the key constraint for adsorption efficiency.

**Figure 3:**
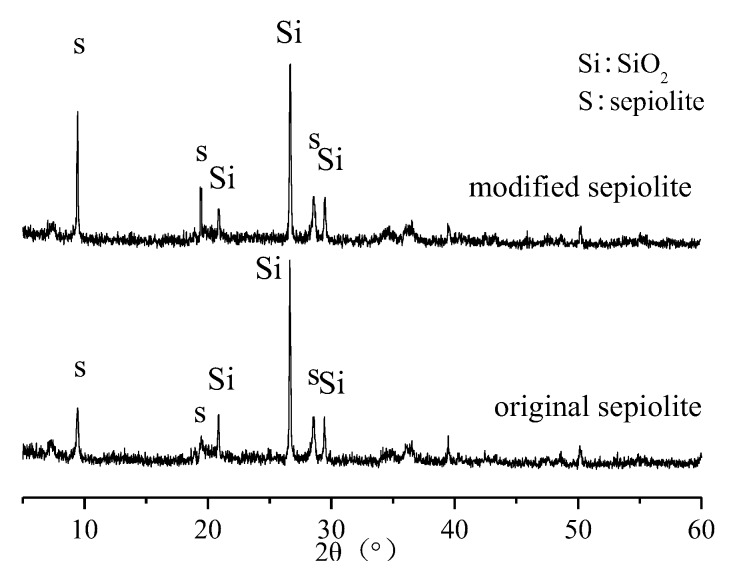
X-ray diffraction spectrum of original sepiolite and modified sepiolite.

As observed from Figure 3, the characteristic peak of the original sepiolite at the 2θ value of 9.4 is the dispersion peak, which obviously enhanced after organic modification. At the same time, the diffraction peaks of quartz were weak after organic modification, but the other characteristic peaks were basically unchanged. This indicates that the SDBS modification of the sepiolite ore did not change its original crystal structure, but the impurity content was reduced and the purity was improved. In addition, the modified sepiolite d_001_ value increased from 1.04 to 1.92 nm. This suggest that the interlayer expanded after the entering of SDBS.

**Figure 4:**
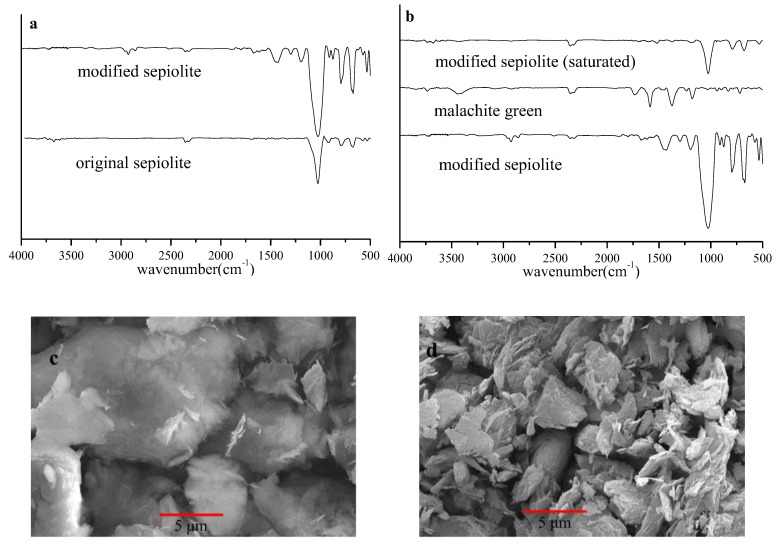
FTIR spectrum of original sepiolite, modified sepiolite, malachite green and modified sepiolite after adsorption saturation, (**a**) and (**b**). SEM images of original sepiolite (**c**) and modified sepiolite (**d**).

Figure 4a presents that characteristic absorption peak of sepiolite, including the Si–O based stretching vibration (1025.8 cm^−1^), the Si–O bending vibration (796.8.8 cm^−1^) and the Mg–O stretching vibration (686.3 cm^−1^). It is indicated that the modification process did not change its original morphology from the crystal structure. Compared with the sepiolite ore, the infrared spectrum of the organically modified organic sepiolite changed as follows: CH bond stretching oscillation belonging to SDBS was 2923 cm^−1^, asymmetric stretching vibration of CH_3_ and CH_2_ were 1440 cm^−1^, 1302 cm^−1^ and 1191 cm^−1^, and the sepiolite characteristic peaks were strengthened. This indicates that the SDBS molecule successfully entered the sepiolite layer, which was consistent with the results obtained by X-ray diffraction analysis. When comparing the FTIR spectra before and after adsorption (Figure 4b), the malachite green characteristic absorption peak appeared on the sepiolite spectrum after adsorption. This is because the surface adsorption/distribution occurred during the adsorption process [21].

The morphology images also confirm that the modification process retained the original crystal structure of sepiolite. Much loose structure was obtained after the modification. Moreover, the interval was obviously variable and smooth. This may be due to the fact that SDBS is a long-chain anionic sulfonate molecular surfactant that formed a negatively charged protective layer on the surface of the sepiolite after ionization, which not only repelled the proximity of the surrounding fibers but also prevented interparticle interaction force. It can be seen that the modification not only retained the original morphology of the sepiolite but also expanded its cross-section and volume.

### 3.3. Impact of the Operation Condition of Adsorption Performance

**Figure 5:**
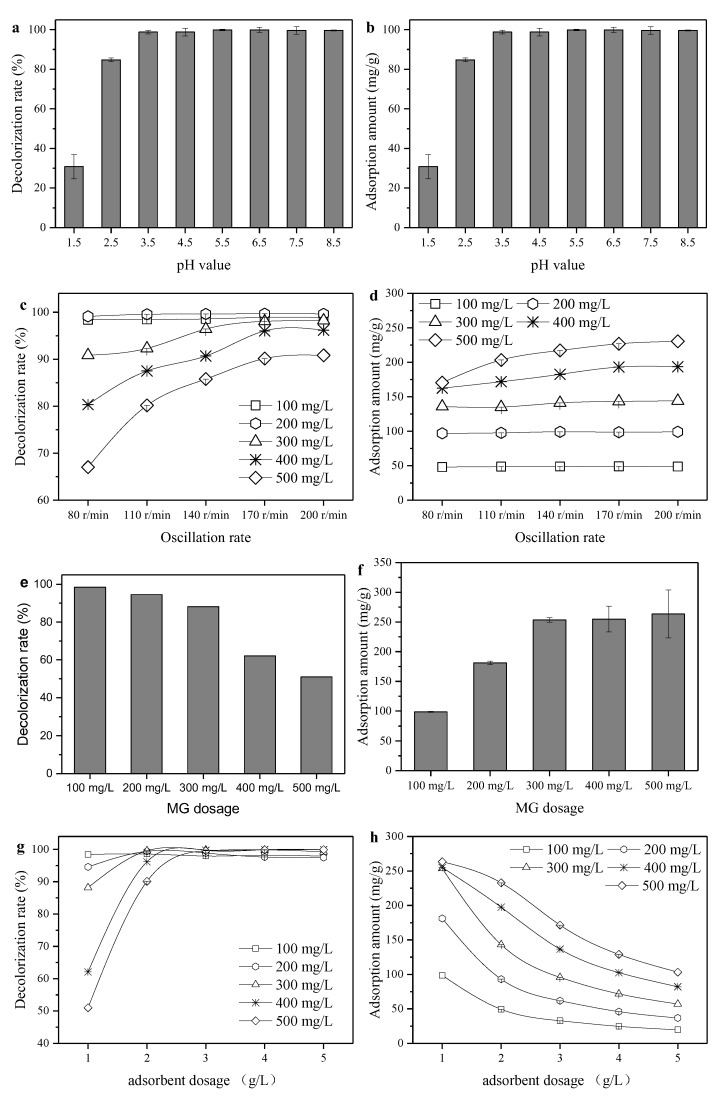
Impact of pH, oscillation rate, MG dosage and adsorbent dosage on adsorption performance. In (**a**) and (**b**), the adsorption test was conducted with a 300 mg/L MG solution and 2 g/L modified sepiolite for 120 min under an oscillation rate of 170 r/min and a temperature of 25 °C; in (**c**) and (**d**), the adsorption test was conducted for 120 min with 2 g/L of modified sepiolite under an initial pH value of 9 and a temperature of 25 °C; in (**e**) and (**f**), the adsorption test was conducted for 120 min with 2 g/L of modified sepiolite under an initial pH value of 9, an oscillation rate of 170 r/min, and a temperature of 25 °C; in (**g**) and (**h**), the adsorption test was conducted for 120 min with an oscillation rate of 170 r/min under an initial pH value of 9 and a temperature of 25 °C.

As can be seen from Figure 5a,b, the decolorization rate improved when the pH value increasing. The H^+^ could affect the surface charge of the adsorbent and the charge distribution in the solution, and it could compete with cationic dye for the adsorption activity of organic sepiolite [22]. Moreover, the positive charge on the surface of the adsorbent could be electrically repelled with the malachite green cation, which was not conducive to the adsorption. The decolorization rate reached 98.78%, and the adsorption amount reached to 144.6 mg/g with a pH value of 3.5. When the pH value continued to increase, the enhancement of adsorption capacity was tiny.

When the concentrations of the dye solution were 100 and 200 mg/L, the oscillation rate had little effect on the decolorization rate, which indicates the good adsorption performance of the organic sepiolite. However, when the dye solution was 500 mg/L, the decolorization rate improved from 67% to 90% with the oscillation rate increasing from 80 to 200 r/min. It can be concluded that the oscillation rate had a great influence during a high dye concentration adsorption process.

The decolorization rate of the dye decreased, but the corresponding adsorption amount increased when the initial dye concentration was increased. In the case where the amount of organic sepiolite was fixed, the organic sepiolite had a large amount of silicic hydroxyl groups and unsaturated ions, and the channel effect was also obvious with the small amount of MG concentration. When the silicic hydroxyl groups and unsaturated ions of the organic sepiolite reached saturation, the adsorption tended to balance, and the surface free energy gradually decreased with an excessive dye solution. The improvement of adsorption amount with higher dye concentrations was probably due to the accumulation of dye on the surface and pores [23].

The decolorization rate of dye liquor increased, but the adsorption amount decreased accordingly when the sepiolite dosage increased. For the high concentration malachite green dyeing solution, the decolorization rate increased with the increase of the amount of the adsorbent, and the adsorption amount decreased. For instance, the MG removal rate increased from 51% to 99%, while the adsorption amount was significantly reduced from 263 to 103 mg/g with an initial dye solution of 500 mg/L.

### 3.4. Adsorption Thermodynamics and Adsorption Kinetics

**Figure 6:**
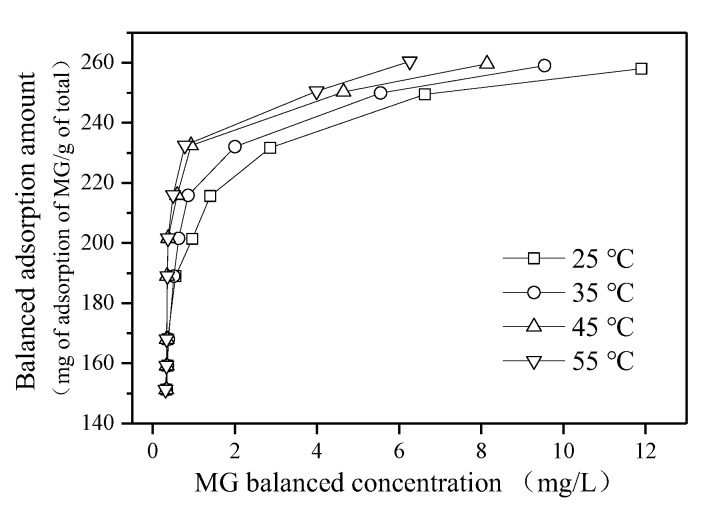
Adsorption isotherm of malachite green. To ensure the adsorbed saturation, the samples were oscillated for at least 10 h at a certain temperature in a constant temperature oscillating box.

As can be observed in Figure 6, the adsorption amount of malachite green improved with the increase of temperature, and the saturated adsorption capacity of organic sepiolite to malachite green dye at 25, 35, 45, and 55 °C were 256.4, 266.7, 270.3, and 277.8 mg/g, respectively.

Table 1 and Table 2 show the Langmuir linear fitting and Freundlich linear fitting, respectively. The linear correlation coefficient of the equation fitting was R_1_^2^ > 0.99 > R_2_^2^, and the theoretically derived single-layer saturated adsorption amount with the Langmuir adsorption isotherm was very close to the experimental. Taking the temperature of 25 °C as an example, the calculated single-layer saturated adsorption amount was 256.4 mg/g and the maximum balanced adsorption amount was 258.0 mg/g. The single-layer saturated adsorption amount q_m_ and the equilibrium constant b increased with the increasing temperature, indicating that an endothermic process occurred and the adsorption capacity enhanced when the tempareture increased [24]. Table 3 also shows the comparison of q_m_ from this work and reported adsorbents. It was noted that SDBS modified sepiolite was also entitled to a high monolayer adsorption capacity, especially considering its low raw material price.

In the absence of interferences, adsorption generally has a negative effect on temperature, because adsorption is an exothermic process. However, the adsorption in this experiment was an endothermic process, presumably because organic sepiolite first desorbed water molecules and then adsorbed malachite green cationic groups in the process of adsorbing malachite green. The process of desorption is an endothermic process, while adsorption is an exothermic process. Since the molar volume of water is much smaller than the molar volume of malachite green dye, the adsorption of a cationic dye required the desorption of a plurality of water molecules, so that the amount of heat required for desorption was greater than the adsorption process. On the other hand, the chemical adsorption probably dominated during adsorption since chemical adsorption is mainly endothermic [26]. The adsorption free energy from Table 4 was from −37.46 to 42.24 kJ/mol, which indicates the adsorption force may have contained electrostatic attraction, Si–OH group attraction, hydrogen bonds, an N atom in the cationic dye skeleton, and Van der Waals force.

**Figure 7:**
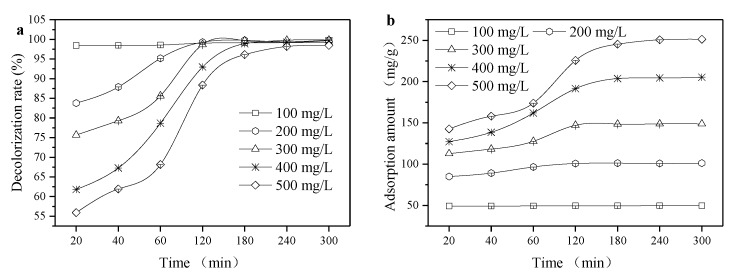
Effect of initial dye concentration on the decolorization rate (**a**) and adsorption amount (**b**). The adsorption test was conducted with 2 g/L of modified sepiolite under an oscillation rate of 170 r/min and a temperature 25 °C.

Figure 7 shows that the adsorption amount of organic sepiolite was gradually increased with the increase of the initial dye concentration. The decolorization curves were nearly flat over 180 min. Therefore, the adsorption process can be divided into two stages. In the initial stage, the adsorbent provided enough adsorption sites and the adsorption rate was fast. Then, the malachite green molecules may have diffused into the interior of the organic sepiolite in the second stage.

When the concentration increased from 100 to 500 mg/L, *k*_1_ increased from 0.6 × 10^−3^ to 23.2 × 10^−3^, and *k*_2_ increased from 0.947 × 10^−4^ to 2.197 × 10^−3^ (Table 5). This suggests that the adsorption driving force caused by the larger concentration gradient was enhanced to facilitate the adsorption procedure. The correlation coefficient of the quasi-first-order reaction rate equation was relatively low (between 0.8183 and 0.9872). However, the correlation coefficient of the quasi-secondary reaction kinetic equation was over 0.99, which indicates that the adsorption of malachite green by organic sepiolite was more in accordance with the quasi-secondary kinetic model. The rate-limiting factor assumed by quasi-secondary reaction kinetics was consistent with the results obtained by thermodynamic analysis. Moreover, the intraparticle diffusion was probably not a control factor during the adsorption process [13].

**Figure 8:**
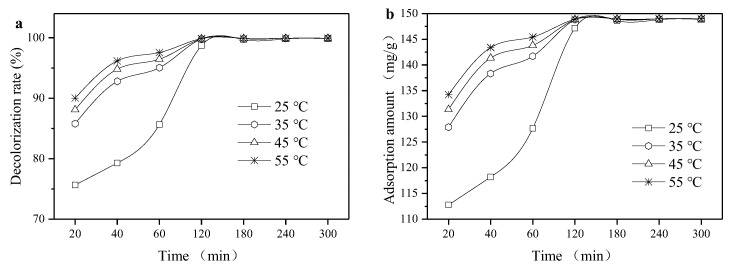
Effect of temperature condition on the decolorization rate (**a**) and adsorption amount (**b**). The adsorption test was conducted with a 300 mg/L MG solution and 2 g/L of modified sepiolite under an oscillation rate of 170 r/min.

Figure 8 shows that equilibrium could be reached around 120 min irrespective of the temperature condition. Therefore, the adsorption process can also be divided into two stages. With the increase of temperature, the adsorption of malachite green by organic sepiolite increased. Under the temperatures of 25, 35, 45, and 55 °C, the equilibrium adsorption amounts of the corresponding organic sepiolite to malachite green were 148.85, 148.97, 148.98, and 148.99 mg/g, respectively.

The *k*_1_ raised from 22.2 × 10^−3^ to 26.7 × 10^−3^, and *k*_2_ raised from 7.3 × 10^−4^ to 3.5 × 10^−3^ with the increasing of temperature (Table 6). The correlation coefficient of the quasi-first-order reaction rate equation was between 0.8840 and 0.9367, which was much below the of the quasi-secondary reaction kinetic correlation coefficient (from 0.9994 to 1). The intraparticle diffusion model shows that this adsorption process had intraparticle diffusion but was not a control step.

### 3.5. Regeneration of Organic Sepiolite Adsorbent

With the increase of the HCl concentration, the regeneration ratio was first raised and then decreased. The regeneration ratio reached 73.56% when 0.2 mol/L HCl applied. During the strong acid regeneration process, the H^+^ was smaller than the dye molecule and could compete with the malachite green cation. Thereby the dye ions were desorbed into the solution. However, the HCl caused the collapse of the sepiolite crystal structure with the high H^+^ concentration [27]. The number of regenerations may reflect the utilize cost of an adsorbent. It can be seen from Table 7 and Table 8 that the decolorization ability of organic sepiolite decreased with the increase of regeneration times. When the number of regenerations reached eight times, the decolorization rate was only 10%. The regeneration test shows that the organic sepiolite can be reused, which could reduce the treatment cost of malachite green wastewater [28].

## 4. Conclusions

In this study, the optimization of sepiolite modification with SDBS, thermodynamics, kinetics analysis and adsorbent regeneration, were comprehensively investigated. The adsorption amount of modified sepiolite with the preferred procedure had a superior result, indicating an economical adsorbent for dye wastewater treatment. The adsorption capacity of modified sepiolite was enhanced though the specific surface area reduction. The characterization suggests that the original crystal structure was retained and a loose structure was obtained. The adsorption tests conform to the Langmuir adsorption isotherm rather than Freundlich adsorption isotherm, thus indicating that an endothermic process occurred. Intraparticle diffusion also occurred during this adsorption process. The adsorption was more in line with the quasi-secondary reaction kinetics model, and chemisorption was the dominated adsorption force.

## Figures and Tables

**Table 1:** Langmuir linear fitting results of organic sepiolite adsorption of malachite green.

Temperature (°C)	Regression Equation	$R_{1}{}^{2}$	$q_{m}\;(\mathrm{mg}/g)$	b (L/mg)
25	C_e_/q_e_ = 0.0039 C_e_ + 0.001	0.9976	256.4	3.9
35	C_e_/q_e_ = 0.00375 C_e_ + 0.0008	0.9998	266.7	4.7
45	C_e_/q_e_ = 0.0037 C_e_ + 0.0007	0.9997	270.3	5.3
55	C_e_/q_e_ = 0.0036 C_e_ + 0.0006	0.9993	277.8	6.1

**Table 2:** Freundlich linear fitting results of organic sepiolite adsorption of malachite green.

Temperature (°C)	Regression Equation	$R_{2}{}^{2}$	k	n
25	ln q_e_ = 0.1409 ln C_e_ + 5.2632	0.9172	193.098	7.097
35	ln q_e_ = 0.146 C_e_ + 5.2901	0.8506	198.383	6.849
45	ln q_e_ = 0.1351 C_e_ + 5.3297	0.7075	206.376	7.402
55	ln q_e_ = 0.1439 C_e_ + 5.3458	0.6952	209.726	6.949

**Table 3:** Comparison of previous study for the removal of MG dyes from aqueous medium.

Adsorbent	Experimental Conditions	q_m_, (mg/g)	Ref.
Fe_3_O_4_@AMCA-MIL53(Al)	*C*_0_: 25–400 mg/L; pH 6.8; T: 45 °C; m: 20 mg	329.61	(Alqadami et al., 2018) [25]
MIL-53(Al)-NH_2_	*C*_0_: 5 mg/L; T: 35 °C; *m*: 10 mg	164.9	(Li et al., 2015) [18]
This work	*C*_0_: 500 mg/L; T: 45 °C; *m*: 10 mg	270.3	

**Table 4:** Fitting results of organic sepiolite adsorption thermodynamic function.

Temperature (K)	b(L/mg)	$b\times 10^{3}\left(L/mol\right)$	ΔH(kJ/mol)	ΔG(kJ/mol)	ΔS(J/mol⋅K)
318	3.90	1423.19	11.50	−37.46	153.96
328	4.69	1710.56	11.50	−39.14	154.39
338	5.29	1928.86	11.50	−40.67	154.35
348	6	2189.52	11.50	−42.24	154.42

**Table 5:** Effect of initial dye concentration on the adsorption kinetics model. The adsorption test was conducted with 2 g/L of modified sepiolite under an oscillation rate of 170 r/min and a temperature of 25 °C.

*C*_0_ (mg/L)	Quasi-First-Order Reaction Kinetics Model			Quasi-Secondary Reaction Kinetics Model			Intraparticle Diffusion Model	
	$q_{1e}$ (mg/g)	$k_{1}$ (1/min)	$R_{1}{}^{2}$	$q_{2e}$ (mg/g)	$k_{2}$ (1/min)	$R_{2}{}^{2}$	$k_{p}$ (1/min)	$r^{2}$
100	2.72	0.6 × 10^−3^	0.8319	49.75	0.947 × 10^−4^	1	0.0339	0.9027
200	13.05	15 × 10^−3^	0.8183	103.09	0.895 × 10^−4^	0.9999	1.2067	0.7663
300	51.04	22.2 × 10^−3^	0.9367	149.25	0.731 × 10^−4^	0.9994	0.9196	0.6648
400	145.98	21.8 × 10^−3^	0.9872	222.22	2.109 × 10^−3^	0.9989	6.521	0.9005
500	270.10	23.2 × 10^−3^	0.9809	277.78	2.197 × 10^−3^	0.9974	9.4062	0.9356

**Table 6:** Effect of temperature on the adsorption kinetics model. The adsorption test was conducted with a 300 mg/L MG solution and 2 g/L of modified sepiolite under an oscillation rate of 170 r/min.

Temperature (°C)	Quasi-First-Order Reaction Kinetics Model			Quasi-Secondary Reaction Kinetics Model			Intraparticle Diffusion Model	
	$q_{1e}$ (mg/g)	$k_{1}$ (1/min)	$R_{1}{}^{2}$	$q_{2e}$ (mg/g)	$k_{2}$ (1/min)	$R_{2}{}^{2}$	$k_{p}$ (1/min)	$r^{2}$
25	51.04	22.2 × 10^−3^	0.9367	149.25	7.3 × 10^−4^	0.9994	0.9196	0.6648
35	149.01	24.3 × 10^−3^	0.8840	151.51	2 × 10^−3^	0.9999	1.1494	0.7108
45	149.11	24.8 × 10^−3^	0.8950	152.67	2.7 × 10^−3^	1	1.4461	0.7542
55	149.23	26.7 × 10^−3^	0.9092	153.85	3.5 × 10^−3^	1	3.0565	0.8563

**Table 7:** Effect of HCl concentration on regeneration ratio.

HCl Concentration (mol/L)	Decolorization Ratio (%)
0	27.14
0.05	33.64
0.1	50.34
0.2	73.56
0.3	60.28
0.5	40.38
1.0	30.88

**Table 8:** Effect of number of regenerations on regeneration ratio under a 0.2 mol/L HCl concentration condition.

Numbers of Regeneration	Decolorization Ratio (%)
0	99.34
1	73.56
2	62.08
4	48.56
6	18.15
8	10.58
10	10.05
